# The impact of urodynamics on treatment and outcomes in women with an overactive bladder: a longitudinal prospective follow-up study

**DOI:** 10.1007/s00192-017-3414-4

**Published:** 2017-07-18

**Authors:** Tina Sara Verghese, Lee J. Middleton, Jane P. Daniels, Jonathan J. Deeks, Pallavi Manish Latthe

**Affiliations:** 1 Institute of Metabolism and System Research, College of Medical & Dental Sciences, University of Birmingham, Birmingham, B15 2TT UK; 2 Birmingham Clinical Trials Unit, University of Birmingham, Birmingham, UK; 3 Birmingham Women’s NHS Foundation Trust, Birmingham, UK

**Keywords:** Urodynamics, Overactive bladder, Detrusor overactivity

## Abstract

**Introduction and hypothesis:**

The aim of this study was to establish if the management of women with overactive bladder (OAB) and patient-reported outcomes differed based on the findings of urodynamics (UDS).

**Methods:**

A prospective, longitudinal observational study conducted in urogynaecology clinics in 22 UK hospitals participating in the Diagnostic Accuracy of Bladder Ultrasound Study (BUS). A total of 687 women with OAB symptoms or urgency-predominant mixed urinary incontinence were recruited into a diagnostic study that used UDS as the reference standard. Detailed clinical history and International Consultation on Incontinence OAB Short Form (ICIQ-OAB sf) questionnaire responses were obtained before the UDS test was carried out. These questionnaires were subsequently collected at a mean of 7 and 20 months, along with patient global impression of improvement and details on medical and surgical treatments. The relationship between UDS diagnosis and treatment was examined using a multinomial regression model; logistic and repeated measures regressions were used to examine other outcomes.

**Results:**

We recruited 687 women and the response rate was 69% at 20 months. Treatment subsequent to UDS was highly associated with diagnosis (*p* < 0.0001). Women who received treatment concordant with their UDS findings were more likely to report an improvement in bladder symptoms (57% vs 45%; *p* = 0.02) and ICIQ-OAB sf scores (0.5 points, 95%CI: 0.1 to 0.9; *p* = 0.02).

**Conclusions:**

Urodynamics influenced treatment decisions made by clinicians in determining treatment pathways in women presenting with OAB. Women treated based on UDS diagnoses appear to have greater reductions in symptoms than those who do not.

**Electronic supplementary material:**

The online version of this article (doi:10.1007/s00192-017-3414-4) contains supplementary material, which is available to authorized users

## Introduction

Urinary incontinence can seriously influence the physical, psychological, and social wellbeing of affected individuals [1]. Urodynamics (UDS) has been considered the test of choice for lower urinary tract symptoms [2]. The International Continence Society (ICS) states that the objective of urodynamic studies is to replicate the patient’s symptoms whilst making measurements that aim to determine the underlying cause and in addition evaluate the related pathophysiological processes [2]. Overactive bladder (OAB) is defined as a symptom complex of urinary urgency (an intense, sudden desire to void) with or without incontinence, usually with increased urinary frequency, or nocturia, but in the absence of infection or other proven pathological condition. From UDS testing, multiple diagnoses may be obtained, including detrusor overactivity (DO), urodynamic stress incontinence (USI), and voiding dysfunction (VD) [3, 4]. In women who present with OAB, 46% do not have a DO diagnosis on UDS [4]. UDS may falsely miss DO or it may not be able to capture DO at that time point [5]. The National Institute of Health and Care Excellence Clinical Guidelines on urinary incontinence (NICE CG171) recommends that patients undergo UDS if they are unresponsive to conservative therapies [1]. There is a dilemma for clinicians as to whether to treat women based on their symptoms or their UDS findings [6–8]. Typically in the UK, patients with OAB are initially offered behavioural therapies and antimuscarinic drugs. NICE guidelines do not recommend performing UDS to commence conservative measures. However, botulinum toxin A can only be offered to women who have not responded to conservative measures and have proven DO on UDS [1]. Invasive therapies (botulinum toxin type A, neuromodulation) can be offered to those with confirmed DO.

There are few data on the longitudinal follow-up of women with urgency or urge-predominant mixed urinary incontinence (MUI) and their response to various medical and surgical therapies [9]. There is a necessity to establish the role of UDS and its impact on treatment and patient outcomes in OAB, as at present its role is unclear.

The aim of this prospective cohort study was to establish if treatment pathways and outcomes differed following findings on UDS. More specifically, we wanted to answer the following questions:

1. Does the UDS diagnosis affect the management offered?
2. What are the long-term clinical outcomes in this group of women as measured by a patient global impression of improvement (PGI-I) question and the International Consultation on Incontinence overactive bladder short form (ICIQ-OAB sf) questionnaire?
3. Does the diagnosis by UDS have any prognostic value for symptoms after 6 and 12 months, i.e. can UDS predict improvement?
4. Does receiving a concordant medical or surgical treatment based on urodynamic diagnoses improve women’s symptoms?

## Materials and methods

**Fig. 1:**
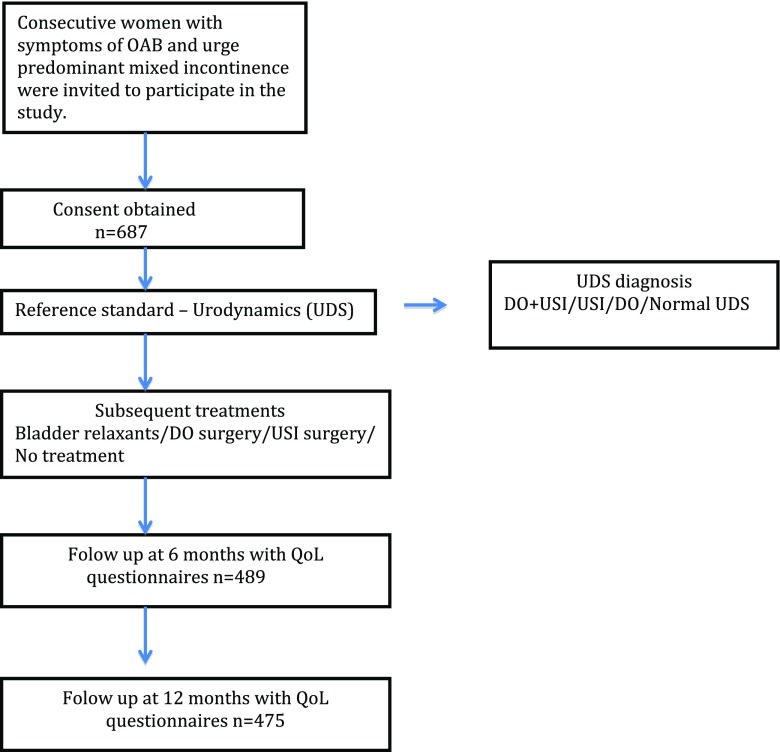
Study flow diagram.* OAB* overactive bladder,* DO* detrusor overactivity,* USI* urodynamic stress incontinence,* QoL* quality of life,

Women who presented consecutively to the urogynaecology units in 22 centres in the UK were recruited into a prospective multicentre study to assess the diagnostic accuracy of bladder ultrasound (BUS study) in diagnosing detrusor overactivity (Fig. 1) [10]. The study was conducted based on good clinical practice guidelines and ethics approval was obtained (Nottingham Research Ethics committee REC: 10/H0408/57).

Women over 18 years of age with symptoms of frequency, urgency, and urge-predominant MUI were approached to participate in the study. Following consent, demographics and detailed clinical history were obtained [10]. Women completed the ICIQ-OAB sf [11] to determine the extent of the impact of their symptoms on quality of life (QoL). All women underwent UDS, using a standardized protocol based on the good urodynamic practice guidance [2]. Questionnaire booklets were sent to women beyond 6 and 12 months post-test. This included a patient PGI-I [12], the ICIQ-OAB sf questionnaire and questions on medical and surgical treatments received by the patients. If no response was received within 4 weeks, reminders were sent. Women who did not respond within 8 weeks of the initial request were contacted by telephone and questionnaires were completed in a telephone interview with a member of the research team where possible.

With the aim of informing a cohesive analysis, we categorized diagnoses from UDS as follows: DO; DO plus USI; USI alone; and women with no demonstrable findings on UDS were defined as normal UDS. In a similar fashion, subsequent treatment data were categorized to be concordant with diagnoses as follows:

1. “DO surgery” for diagnosis including DO, percutaneous tibial nerve stimulation (PTNS) or sacral neuromodulation and/or botulinum A toxin injections into the bladder.
2. “USI surgery” for diagnosis including USI, midurethral sling including tension-free vaginal tapes (retropubic or transobturator), fascial sling, bladder neck injection and colposuspension.
3. “Bladder relaxants”—antimuscarinics and Mirabegron use were considered concordant with a UDS diagnosis including DO or normal UDS
4. “No treatment” was considered concordant with a UDS diagnosis of normal UDS

Other assumptions were that the effect of bladder relaxants was assumed only at the time at which it was reported, whereas the effect of surgery was assumed to be longer (i.e. those reporting surgery at 6 months were presumed to still have had surgery at 12 months). In addition, the women who reported that they had had both surgery and bladder relaxants as a proportion of those having surgery were in the minority (15% at 6 months, 26% at 12 months). Surgery was assumed to supersede the use of bladder relaxants, so to simplify the analysis, this dual effect was ignored (i.e. for women categorized as having USI or DO surgery, this may or may not include the use of bladder relaxants).

The relationship between UDS diagnoses and subsequent treatment was examined using a multinomial logistic regression model [13] with the treatment group variable as the outcome and UDS diagnosis as the explanatory variable. The overall importance of this variable was determined using Chi-squared test with results presented alongside estimates of odds ratios (OR) and 95% confidence intervals (CI; the “no treatment” group was used as the reference). Frequencies and percentages are presented for the results of the PGI-I question measured at 12 months, with means and standard deviations presented for ICIQ-OAB scores (measured on a 0 [best] to 16 [worst] scale) after 6 and 12 months. Mean change from baseline and 95% confidence intervals were calculated and paired* t* tests were used to test statistical significance. Further analysis was completed using logistic and linear repeated measures regression models [14] to examine the effect of UDS diagnosis on the patient-reported outcome measures (PROMs) and whether treatment was concordant with UDS diagnoses. The statistical importance of these explanatory variables was determined using Chi-squared and* t* tests. Comparative odds ratios (OR) were calculated along with 95% CI using the standard error (SE) taken from the respective models.

## Results

The study recruited a total of 687 women, with a mean age of 52.7 years (SD 13.9) and an average body mass index of 30.6 (SD 12.2). Fifty-five percent of the women (387 out of 687) were postmenopausal. According to clinical history, 61% (419 out of 687) had urgency-predominant MUI and 33% (226 out of 687) reported urinary urgency along with increased frequency. The median duration of symptoms was 3.0 years (IQR: 1.6, 7.0). Out of 687 women recruited, 97% (666) had complete UDS test results. On UDS, 43% (*n* = 284) were diagnosed with DO alone, 17% (*n* = 115) had DO + USI, 14% (*n* = 92) were diagnosed with USI alone and 26% (*n* = 175) had normal UDS. The questionnaire response rates were 489 (71%) and 475 (69%), received at a median time of 7 and 20 months following testing (results are referred to below as being at these times).

### Effect of UDS on the management offered

Over the whole follow-up period, 63% of the women (292 out of 467) reported that they had some form of condition-related treatment; 70% of these treatments (205 out of 292) were reported as bladder relaxants only, 20% USI surgery (57 out of 292) and 10% DO surgery (30 out of 292; Table 1). Overall, subsequent treatment was found to be highly associated with diagnosis group (*p* < 0.0001); the odds of treatment for each diagnosis (using the normal UDS diagnosis group as a reference) are given in Table 2.

**Table 1:** Reported interventions over the whole follow-up period by UDS diagnosis: treatment frequency indicated

	Treatment				
UDS diagnosis	DO surgery (plus bladder relaxants)	USI surgery (plus bladder relaxants)	Bladder relaxants only	No treatment	Total
DO + USI	3 (3)	27 (11)	29	23	82
DO	19 (5)	6 (3)	119	57	201
USI	2 (0)	18 (6)	16	25	61
Normal UDS	6 (1)	6 (1)	41	70	123
Total	30	57	205	175	467^a^

*UDS* urodynamics,* DO* detrusor overactivity,* USI* urodynamic stress incontinence

^a^8 participants returned follow-up forms but did not complete treatment information

**Table 2:** Odds ratios (OR) of intervention versus no treatment over the whole period using the normal UDS diagnosis group as a reference

	Treatment		
Diagnosis effect	DO surgery OR (95%CI)	USI surgery OR (95%CI)	Bladder relaxants OR (95%CI)
DO vs normal UDS	2.6 (1.2, 5.4)	1.0 (0.3, 2.7)	3.3 (2.4, 4.7)
DO + USI vs normal UDS	0.8 (0.2, 2.8)	14.9 (6.6, 33.8)	2.0 (1.2, 3.2)
USI vs normal UDS	1.0 (0.3, 3.2)	8.2 (3.5, 19.3)	1.1 (0.7, 1.9)

### Medium-term response in the entire cohort

Fifty-three percent of the women (248 out of 470) considered their bladder problems to have improved (PGI-I responses) at 20 months. ICIQ scores also improved on average, reflecting the declining severity of women’s symptoms (Table 3).

**Table 3:** International Consultation on Incontinence Questionnaire (ICIQ) scores from baseline to 20 months

	Mean (SD),* n*	Change from baseline: mean, 95%CI, *p* value
Baseline	9.3 (2.7), 637	
7 months	7.3 (3.3), 469	−1.9 (−2.2, −1.6), < 0.0001
20 months	7.0 (3.5), 460	−2.2 (−2.5, −1.9), < 0.0001

### Medium-term responses by UDS diagnoses

The proportion of women indicating improvement on the PGI-I question was higher in the USI (63%; 35 out of 56) and DO + USI (58%; 48 out of 83) groups compared with the DO (51%; 104 out of 205) and normal UDS (48%; 60 out of 125) groups, although not enough to be statistically significant (*p* = 0.2). ICIQ scores were reduced from baseline in all groups at both time-points (*p* < 0.001; Table 4, Fig. S1). There was some evidence that ICIQ responses varied between diagnosis groups (*p* = 0.02); pairwise comparisons indicated that the DO + USI group had a greater reduction than the DO group (−1.1 points, 95% CI: −1.7, −0.4; *p* = 0.002) overall. There were no statistically significant differences between the other diagnosis groups.

**Table 4:** ICIQ scores by diagnosis groups

	UDS diagnosis			
	DO + USI	DO	USI	Nothing
Baseline mean (SD),* n*	9.8 (2.5)	9.8 (2.7)	8.9 (2.7)	8.2 (2.3)
7 months mean (SD),* n*	6.8 (3.5), 81	8.1 (3.4), 207	7.1 (3.3), 57	6.5 (2.8), 124
20 months mean (SD),* n*	6.6 (3.6), 82	7.7 (3.8), 199	6.2 (3.3), 55	6.5 (3.1), 123

### Effect of receiving a medical or surgical treatment concordant with a UDS diagnosis

At 20 months, 57% of women (168 out of 296) who had received a treatment concordant with diagnosis felt that they had improved, as opposed to only 45% (69 out of 152) in those who had not (OR: 1.6, 95% CI 1.1 to 2.3; *p* = 0.02); there was no overall evidence that this varied by UDS diagnosis (*p* = 0.1; Table 5). ICIQ scores were reduced at 7 and 20 months, regardless of whether the patient had had a concordant treatment or not, although the scores were better in those who did (−0.5, 95%CI: −0.9, −0.1; *p* = 0.02). There was no evidence that the effect of receiving a concordant treatment varied by UDS group (*p* = 0.3; Table S1).

**Table 5:** Proportion of patients reporting improvement in symptoms by UDS diagnosis and whether a medical or surgical treatment concordant with this diagnosis had been received

UDS diagnosis	Concordant medical or surgical treatment = no (%)	Concordant medical or surgical treatment = yes (%)	OR (95%CI)
DO + USI	8/25 (32)	39/55 (71)	5.2 (1.9, 14.4)
DO	36/79 (46)	63/118 (53)	1.4 (0.8, 2.4)
USI	24/41 (59)	10/14 (71)	1.8 (0.5, 6.6)
Normal UDS	1/7 (14)	56/109 (51)	6.3 (0.7, 54.4)
Total	69/152 (45)	168/296 (57)	

## Discussion

### Main findings and interpretation

Urodynamics appears to influence treatment decisions made by clinicians and patients in determining treatment pathways in women presenting with OAB. Women with DO were three times more likely to have had bladder relaxants than no treatment than women with a normal diagnosis. This could be interpreted as those who were shown to have DO either received prescribed bladder relaxant tablets more or patient compliance with taking the treatment was better. Women with a diagnosis of DO plus USI were 15 times more likely to have USI surgery than no treatment, which may at least partly explain the improved ICIQ scores and PGI-I in this group compared with the pure DO group.

A multicentre randomised double-blind trial (RCT) to determine whether women with or without a UDS finding of DO responded differently to antimuscarinic treatment demonstrated that UDS status was unable to predict treatment outcomes in women treated with the antimuscarinic agents or placebo [11]. The objective in a recent Cochrane systematic review [15] was to determine if treatment according to UDS-based diagnosis compared with treatment based on history and examination alone led to more effective clinical care and better clinical outcomes in women with urinary incontinence. Two of the included trials [16, 17] demonstrated that women who underwent UDS were more likely to receive drugs to treat their symptoms than who did not (45% vs 21%, RR 2.09, 95% CI 1.32–3.31). Furthermore, three trials [18–20] found that those who had UDS were more likely to have their management changed (17% vs 3%, RR 5.07, 95% CI 1.87–13.74), although in five trials [16–19, 21], it was found that women were not more likely to undergo surgery after UDS (81% vs 79% RR 0.99, 95%CI 0.88–1.12). The evidence for the included surgical trials was of moderate quality (based on GRADE outcomes). Contrary to the findings of the Cochrane systematic review, we found that more women with DO plus USI diagnosis had received surgery by 20 months’ follow-up compared with those with a normal UDS diagnosis. Confirmation of the concurrent pathophysiology of DO plus USI may have resulted in the more clinicians offering USI surgery after suboptimal improvement with bladder relaxants alone.

In the overall population at 20 months, just over half the women (53%) reported long-term improvement in symptoms and ICIQ scores were reduced from baseline by 2.2 points (*p* < 0.001) on average, a difference that appears to be clinically meaningful. However, women treated with medical or surgical interventions based on UDS diagnoses appear to have greater reductions in symptoms than those who were not (57% vs 45%; *p* = 0.02). ICIQ scores were reduced at both time points regardless of whether the women received a treatment concordant with UDS findings or not, although patients receiving a concordant treatment reported a slightly greater reduction (−0.5 points; *p* = 0.02). The improvement reported by those who did not receive a concordant treatment could be for several reasons, such as the natural fluctuation of the disease state, regression to the mean and Hawthorne effect (individuals modify an aspect of their behaviour as a response to their awareness of being observed) [22]. The experience of UDS may have helped women to understand their condition better and to improve compliance with lifestyle measures and medications.

### Strengths and limitations

This study is one of the few reporting on the prospective follow-up in women with urgency and urge-predominant MUI and reporting better outcomes in the MUI group in comparison to the DO group. The response rate for continued follow-up of the cohort was 69% at 20 months; although not high, it is superior to other studies in the field. We captured the opportunity to assess the prognostic value of UDS and the outcomes following urodynamic diagnoses. The patients were followed up for more than 12 months and validated questionnaires were used. We believe that these data will hopefully offer some evidence to the clinical community that UDS does change patient management in current clinical practice.

The major limitation of this study is that it is not a randomized controlled trial (RCT) of outcomes based on treatment given to those with or without DO. Our results are therefore subject to unknown confounders, which may bias our results, including decisions to treat being based on information from sources other than UDS. Also, we could only ascertain whether women had “ever” having taken bladder relaxants as opposed to women currently taking bladder relaxants. Second, the number of women having both bladder relaxants and surgery was small, and therefore could not be reliably distinguished from those who had surgery alone. In addition, we did not collect data on therapies such as supervised intensive pelvic floor muscle training, bladder retraining, lifestyle changes etc., but we presumed that the conservative treatment was already exhausted before patients were referred for UDS. A further limitation was that we did not link information on HRT use with urodynamic diagnoses. Lack of oestrogen following the menopause is known to cause atrophic changes, which may be associated with lower urinary tract symptoms [23].

The response rate was 69%, in spite of sending reminder questionnaires, emails and telephone calls to improve this yield. Although we have no reason to suspect that patients with missing responses were any different from those who responded to questionnaires we cannot rule out that this may have affected our conclusions to an unknown degree.

## Conclusions

Urodynamics appears to influence treatment decisions made by clinicians in determining treatment pathways in women presenting with OAB. Women treated based on UDS diagnoses appear to have greater reductions in symptoms than those who do not. In women with OAB, a multicentre RCT comparing patient outcomes of treatment based on UDS diagnoses versus treatment based on clinical assessment (history and examination alone) and related health economic evaluation for these diagnostic interventions is planned. This will help to consolidate the role of UDS in the management of these women, as has been done for stress urinary incontinence [24].

## Electronic supplementary material

ESM 1 (DOCX 73 kb)

ESM 2 (DOCX 80 kb)
