# Supplementary material for: The impact of urodynamics on treatment and outcomes in women with an overactive bladder: a longitudinal prospective follow-up study
Source: Int Urogynecol J. 2017 Jul 18;29(4):513–9. doi: 10.1007/s00192-017-3414-4 (PMC5876271; doi:10.1007/s00192-017-3414-4)
Supplement: Supplementary file 1 — (DOCX 73 kb) [file 192_2017_3414_MOESM1_ESM.docx]

**Figure S1: ICIQ scores over time by diagnosis group**


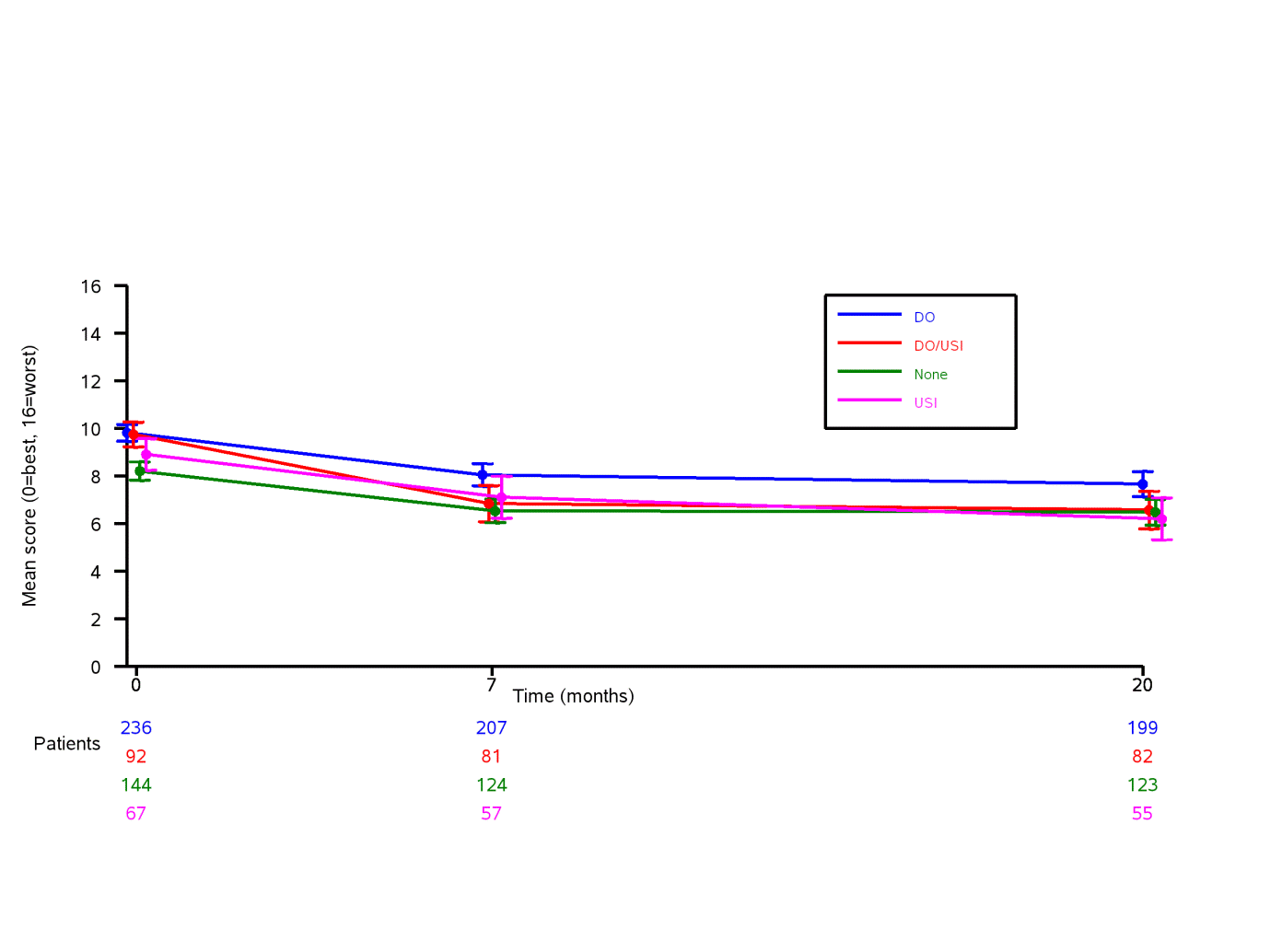


95% confidence intervals are shown at each time point
