# Supplementary material for: The impact of urodynamics on treatment and outcomes in women with an overactive bladder: a longitudinal prospective follow-up study
Source: Int Urogynecol J. 2017 Jul 18;29(4):513–9. doi: 10.1007/s00192-017-3414-4 (PMC5876271; doi:10.1007/s00192-017-3414-4)
Supplement: Supplementary file 2 — (DOCX 80 kb) [file 192_2017_3414_MOESM2_ESM.docx]

**Table S1: ICIQ scores by UDS diagnosis and whether a medical or surgical treatment concordant with this diagnosis had been received**

|  | Concordant medical or  surgical treatment=no | | Concordant medical or surgical treatment=yes | |
| --- | --- | --- | --- | --- |
|  | Mean (SD), n | Change from baseline:  mean, 95%CI, p-value | Mean (SD), n | Change from baseline:  mean, 95%CI, p-value |
| DO + USI |  |  |  |  |
| Baseline | 9.4 (2.1) |  | 9.5 (2.7) |  |
| 7 months | 6.9 (3.2), 28 | -2.7 (-4.0, -1.4), p=0.0003 | 6.2 (3.7), 38 | -3.4 (-4.5, -2.3), p<0.0001 |
| 20 months | 7.4 (3.6), 26 | -1.8 (-3.3, -0.4), p=0.02 | 6.0 (3.4), 53 | -3.6 (-4.6, -2.6), p<0.0001 |
| DO |  |  |  |  |
| Baseline | 9.6 (2.8) |  | 9.9 (2.8) |  |
| 7 months | 8.6 (3.6), 79 | -1.0 (-1.6, -0.4), p=0.002 | 7.8 (3.3), 111 | -2.2 (-2.8, -1.6), p<0.0001 |
| 20 months | 7.6 (3.8), 77 | -1.6 (-2.3, -0.8), p<0.0001 | 7.9 (3.8), 115 | -2.3 (-3.0, -1.6), p<0.0001 |
| USI |  |  |  |  |
| Baseline | 8.9 (2.4) |  | 9.3 (4.0) |  |
| 7 months | 7.1 (3.4), 45 | -1.8 (-2.8, -0.8), p=0.0006 | 6.5 (4.1), 6 | -2.8 (-7.8, 2.1), p=0.2 |
| 20 months | 6.4 (3.3), 40 | -2.6 (-3.8, -1.3), p=0.0001 | 5.8 (3.1), 14 | -2.3 (-4.3, -0.2), p=0.03 |
| NORMAL UDS |  |  |  |  |
| Baseline | 9.9 (2.0) |  | 8.0 (2.3) |  |
| 7 months | 6.3 (3.5), 7 | -3.6 (-6.9, -0.2), p=0.04 | 6.5 (2.8), 106 | -1.5 (-1.9, -1.0), p<0.0001 |
| 20 months | 8.1 (3.2). 7 | -1.7 (-5.1, 1.8), p=0.3 | 6.3 (3.1), 107 | -1.9 (-2.4, -1.3), p<0.0001 |
